# Pan-cellular organelles and suborganelles—from common functions to cellular diversity?

**DOI:** 10.1101/gad.351337.123

**Published:** 2024-02-01

**Authors:** Rico Schieweck, Magdalena Götz

**Affiliations:** 1 Institute of Biophysics, National Research Council (CNR) Unit at Trento, 38123 Povo, Italy;; 2 Biomedical Center (BMC), Department of Physiological Genomics, Ludwig-Maximilians-University, 82152 Planegg-Martinsried, Germany;; 3 Institute of Stem Cell Research, Helmholtz Center Munich, German Research Center for Environmental Health, 82152 Planegg-Martinsried, Germany;; 4 SYNERGY, Excellence Cluster of Systems Neurology, Biomedical Center, Ludwig-Maximilians-University, 82152 Planegg-Martinsried, Germany

**Keywords:** specialized organelles, organellar proteome, RNA–organelle interaction, RNA inheritance, cell fate commitment

## Abstract

Cell diversification is at the base of increasing multicellular organism complexity in phylogeny achieved during ontogeny. However, there are also functions common to all cells, such as cell division, cell migration, translation, endocytosis, exocytosis, etc. Here we revisit the organelles involved in such common functions, reviewing recent evidence of unexpected differences of proteins at these organelles. For instance, centrosomes or mitochondria differ significantly in their protein composition in different, sometimes closely related, cell types. This has relevance for development and disease. Particularly striking is the high amount and diversity of RNA-binding proteins at these and other organelles, which brings us to review the evidence for RNA at different organelles and suborganelles. We include a discussion about (sub)organelles involved in translation, such as the nucleolus and ribosomes, for which unexpected cell type-specific diversity has also been reported. We propose here that the heterogeneity of these organelles and compartments represents a novel mechanism for regulating cell diversity. One reason is that protein functions can be multiplied by their different contributions in distinct organelles, as also exemplified by proteins with moonlighting function. The specialized organelles still perform pan-cellular functions but in a cell type-specific mode, as discussed here for centrosomes, mitochondria, vesicles, and other organelles. These can serve as regulatory hubs for the storage and transport of specific and functionally important regulators. In this way, they can control cell differentiation, plasticity, and survival. We further include examples highlighting the relevance for disease and propose to examine organelles in many more cell types for their possible differences with functional relevance.

## Abstract (precis)

In this review, Schieweck and Götz describe our current understanding of organelle and suborganelle heterogeneity, particularly in their composition, function, and regulation. They further expand on how organelle heterogeneity contributes to cellular diversity and the maintenance of essential cellular functions that are relevant for development and disease.

Since the early days of cell biology, researchers have described the compartmentalization of cells as crucial for their function. These compartments include membranous organelles such as the nucleus, endoplasmic reticulum, and lysosomes, as well as nonmembranous organelles such as centrosomes in the cytoplasm or the nucleolus or speckles in the nucleus (Alberts et al. 2014). These organelles fulfill similar pan-cellular functions within cells, such as the centrosome organizing the cytoskeleton, migration, and cell division, as well as the basal bodies of cilia (Conduit et al. 2015). Likewise, the nucleolus orchestrates translational processes in all cell types. However, both these organelles differ in different cell types, with the centrosome even differing by more than half of the proteome between closely related cell types (Chang and Marshall 2017; Camargo Ortega and Götz 2022; O'Neill et al. 2022). Surprisingly, RNA-binding proteins (RBPs) are among the top categories of proteins that have been detected in numerous organelles and differ at the centrosome of different cell types (O'Neill et al. 2022). Therefore, not only does protein diversity convey distinct functional aspects to these organelles, but specific RBPs at particular organelles also enable the recruitment of specific mRNAs. As a consequence, organelles differ not only in their protein composition but also in their RNA interactors. They provide a platform for controlling RNA transport, localized translation, RNA storage, or localized RNA degradation. It is important to note that the levels of specific RNA and protein interactors vary between different cells (Döhla et al. 2022; O'Neill et al. 2022) and between locations within a single cell (Harbauer et al. 2022). For example, some proteins can be at one localization or organelle in one cell type and at another in another cell type. These moonlighting functions of proteins discussed below contribute to organellar heterogeneity.

Specialized organelles are able to regulate essential cellular functions through interactions with specific proteins and mRNAs. This is particularly important in complex cells with elaborate functional subcompartments, such as neurons or skeletal muscle cells (Schieweck et al. 2021a). Here we provide examples of cell type-specific differences in organelle composition affecting development or reprogramming. This also prompts the topic of the fate of daughter cells that can be influenced by the asymmetric inheritance of factors during cell division. Again, organellar inheritance contributes to the establishment of differences in daughter cell fates, as has been shown for mitochondria inheritance from stem cells (Katajisto et al. 2015; Cheng et al. 2022; Döhla et al. 2022).

Last, we also touch on the relevance for disease. Mutations that affect protein function at a specific organelle can cause developmental defects. Here we discuss how organellar heterogeneity can explain why ubiquitous proteins can cause organ-specific phenotypes when mutated. This is due to their localization at specific organelles only in certain cell types (O'Neill et al. 2022). These examples highlight the urgent need to consider the heterogeneity of pan-cellular organelles at both their protein and RNA levels, and we call for further profiling of different cell types in this regard. We propose the concept that organellar heterogeneity may serve as a means to further diversify the function of these organelles and accordingly expand cell diversity through a novel regulatory layer. This may allow a further increase in cell diversity despite our limited gene numbers in ontogeny and phylogeny and has important implications for disease.

In this review, we discuss our current understanding of organellar heterogeneity at both the protein and RNA interactor levels. Additionally, recent findings on developmental disorders caused by malfunctioning of specialized organelles are highlighted. Finally, a working model is proposed for how heterogeneity of some organelles (namely, the membrane-less organelles) may be established in a cell type-specific manner.

## Unexpected heterogeneity of pan-cellular organelles

Here we focus on reviewing the emerging evidence for organellar differences between cells at both the proteome and RNA level, as much as is known in this new emerging field. We discuss this with a broad scope ranging from proteins at one specific organelle only in specific cell types to proteins present in many cell types but only at a certain organelle in specific cell types. We further touch on heterogeneity of organelles or suborganelles at different locations within a single cell type; for example, axons and dendrites in neurons. In the next section, recent findings are highlighted, showing noncanonical organellar interactors that imply novel functions of these organelles.

### Centrosomes

**Figure 1.**
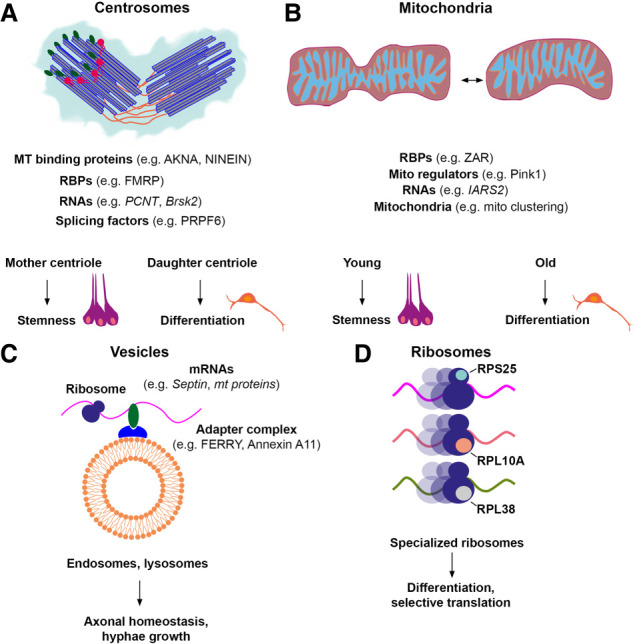
Specialization of pan-cellular organelles. (*A*) Centrosomes consist of an older mother centriole and a younger daughter centriole. These centrioles are duplicated prior to stem cell mitosis and are asymmetrically distributed to the progeny. This has been linked to the maintenance of the stemness of the cell receiving the older mother centriole and can induce differentiation of the daughter cells inheriting the younger centriole. To regulate these processes, centrosomes interact with many proteins, including RBPs, splicing factors, and RNAs. (*B*) New mitochondria are generated by fission events. These young mitochondria accumulate preferentially in cells that maintain their stemness when the mother stem cell divides. In contrast, older mitochondria are inherited by the differentiating progeny. To maintain mitochondrial function, these organelles interact with RBPs and transcripts encoding proteins required for mitochondrial function and mitophagy. (*C*) Vesicles serve as transport platforms for actively translated mRNAs. Therefore, RBPs such as the five-subunit endosomal Rab5 and RNA/ribosome intermediary (FERRY) complex interact with the surface of these vesicles to recruit transcripts. In this way, vesicles distribute mRNAs and newly synthesized proteins to maintain cellular homeostasis. (*D*) Ribosomes are composed of rRNA and ribosomal proteins. The composition of ribosomal proteins is variable. As a result, specialized ribosomes exist that interact with a specific subset of ribosomal proteins to control the translation of specific transcripts.

Centrosomes are well known for their role in acting as cytoskeleton organizing centers for the microtubule (MT) network in migratory cells and for the mitotic spindle in dividing cells. Moreover, it forms the basal body of cilia. As these functions are similar in many different cell types, it came as a surprise when pioneering studies showed that their composition differs hugely even between closely related cell types (O'Neill et al. 2022), such as neural stem cells and neurons that differ by >50% of their comprehensive proteome (Fig. 1A; O'Neill et al. 2022).

Notably, for individual proteins, this difference had already been shown before, such as for the protein Akna, which is at the centrosome in differentiating neural stem cells but not self-renewing neural stem cells (Camargo Ortega et al. 2019), and specific isoforms of Ninein that associate with the centrosome in neural stem cells (NSCs) versus neurons (Zhang et al. 2016). There are also further examples from other tissues in this review (Camargo Ortega and Götz 2022) showing that differentiating cells often lose microtubule-organizing center activity at the centrosome also in tissues other than the brain. The centrosome consists of two centrioles (the mother and daughter centrioles) and the pericentriolar material that are functionally and structurally distinct (Fig. 1A; Tischer et al. 2021). Pull-down experiments using different bait proteins that display distinct centrosomal localizations highlighted the heterogeneity of centrosomes from NSCs and neurons. NSC-specific centrosomal proteins preferentially localize to the appendages of the mother centriole where, for example, MTs are anchored or the membrane contact is established. In contrast, neuron-specific centrosomal proteins mostly interact with Cep63 (O'Neill et al. 2022). The latter is particularly intriguing, as Cep63 has so far been implicated in centriole duplication, which is not relevant in postmitotic neurons. While much remains to be understood about this amazing difference in centrosome composition between neural stem cells, neurons, and other cell types, this work showed the fundamental importance of centrosome localization of proteins regulating RNA, as the top category of proteins with differential centrosome localization between NSCs and neurons was RNA-related (O'Neill et al. 2022). Among them was also an entire splicing complex, comprising the splicing factor, pre-mRNA-processing factor 6 (PRPF6), and its interactors, such as Acin1, DDX23, and KIAA1429 (O'Neill et al. 2022). PRPF6 is expressed in virtually all cells of our body. However, in addition to its localization to the nuclear splicing compartments, it localizes to the centrosome in neural stem cells but not other cell types examined. When this protein is mutated, delamination of differentiating neural stem cells from the stem cell niche at the ventricle is impaired, causing periventricular heterotopia, with some cells stuck at the ventricle. Interestingly, PRPF6 regulates the splicing of *Brsk2* (O'Neill et al. 2022), which encodes the SAD-A kinase that phosphorylates microtubule-associated proteins (MAPs) to control MT dynamics (Kishi et al. 2005; Barnes et al. 2007), and *Brsk2* RNA is localized at the centrosome (O'Neill et al. 2022). *Brsk2* RNA at the centrosome is reduced by cycloheximide but not puromycin treatment, and the SAD-A protein is also present at the centrosome (Antognolli 2024), consistent with local translation. Most importantly, however, the periventricular heterotopia phenotype caused by the mutated PRPF6 can be rescued by supplying the correctly spliced form of *Brsk2* (O'Neill et al. 2022). These data demonstrate that this ubiquitous splicing factor plays an organ-specific role in the brain, correlating to its centrosomal localization and the (local) translation of its splicing targets.

A possible causative role of MT alteration by SAD-A at the centrosome is further supported by the crucial function of MTs at the centrosome for delamination and migration out of the ventricular stem cell niche, as shown by the role of Akna (Camargo Ortega et al. 2019). Akna is a novel centrosome protein (also binding MTs and RNA) (Hoefig et al. 2021) specifically expressed in differentiating neural stem cells. It is necessary and sufficient for MT organization at the centrosome, and reduced levels of Akna also result in the failure of cells to leave the ventricle (Camargo Ortega et al. 2019). Importantly, the key role of Akna at the centrosome was demonstrated by using a truncated protein still binding MTs but no longer the centrosome. This protein could not affect the delamination of the NSCs from the ventricle, while the full-length protein localized to the centrosome had a strong effect on cells leaving the ventricle (Camargo Ortega et al. 2019). Thus, centrosomal MTOC activity mediated by cell type-specific centrosomal localization of Akna has profound functional effects on the fate of neural stem cells, causing them to leave their niche and differentiate.

Another example of a highly cell type-specific centrosome association of an MT binding protein is Ninein. This protein also anchors microtubules to centrosomes, and its loss impairs the maintenance of apical stem cells (Wang et al. 2009, 2020; Shinohara et al. 2013). Interestingly, alternative splicing in neurons causes Ninein to relocalize from centrosomes to microtubules (Zhang et al. 2016). Thus, Ninein is mainly associated with centrosomes in self-renewing neural stem cells, while Akna takes over in differentiating neural stem cells (Camargo Ortega and Götz 2022), highlighting the impressive degree of specificity in protein localization at the centrosome, even differing in subtypes of neural stem cells (Fig. 1A). Moreover, expression of the Ninein splice variant present normally in neurons (i.e., not at the centrosome) promotes delamination of neural stem cells and depletes them at the ventricle, thus showing direct functional consequences for neural stem cell differentiation (Zhang et al. 2016). Taken together, even in the G1 phase when both of these proteins are localized at the centrosome, the specific composition of the centrosome plays important functional roles. Given their abundance as a top GO category of cell type-specific proteins and the crucial function of PRPF6, the role of other centrosome-interacting RNA-binding proteins especially should be examined further and at the functional level.

In this regard, it is important to mention that not only do cells express a large number of RBPs (Castello et al. 2012, 2016; Hentze et al. 2018; Caudron-Herger et al. 2019) with diverse functions (Schieweck et al. 2021b) but these also localize to distinct places in cells. These proteins exhibit a distinct set of mRNA interactors, thereby defining the RNA interactome of organelles. Which RNAs are localized at the centrosome has not yet been determined in a comprehensive manner, but in a screen of ∼100 RNAs encoding centrosome proteins, only a few were found to localize to the centrosome, including *pericentrin* (*Pcnt*) (Safieddine et al. 2021), demonstrating a highly selective process in recruiting only some RNAs to the centrosome. However, a large cell type-specific set of RBPs has been found at the centrosome in neural stem cells (O'Neill et al. 2022), including Staufen and the fragile X mental retardation protein (FMRP) (O'Neill et al. 2022). Pioneering studies have shown that Staufen and its RNA targets are asymmetrically inherited to daughter cells, which define the cell fate of these cells (Li et al. 1997; Kusek et al. 2012; Vessey et al. 2012). It is conceivable that Staufen recruits mRNAs to the centrosome (to the mother or daughter centriole) in neural stem cells, which are then asymmetrically distributed to the progeny during cell division. FMRP plays a crucial role in inhibiting the translation of target RNAs (Chen et al. 2014) and is also required for proper neuronal development (Gao et al. 2018; Edens et al. 2019). Interestingly, FMRP also inhibits the recruitment of mRNAs to the centrosome in *Drosophila* embryos (Ryder et al. 2020). These findings suggest that the RBP interactome determines the centrosomal RNA composition, and future studies are needed to address to what extent this effect is cell type-specific.

Prior to cell division, centrosomes are duplicated, resulting in two centrosomes with differently aged mother centrioles. This process has been implicated in determining the fate of daughter cells. Most neural stem cells in the murine cerebral cortex that inherit new daughter centrioles leave the ventricular stem cell niche to become differentiating progenies (Fig. 1A). In contrast, neural stem cells in the murine cerebral cortex with old mother centrioles remain in the ventricular zone and maintain their original stemness (Wang et al. 2009). The mother centriole is also crucial for forming the basal body of the primary cilium, a short microtubule-based protrusion that is essential for receiving signals. As neural stem cells have their cilium at the apical side sticking into the ventricle filled with cerebrospinal fluid, it can sense signals from the cerebrospinal fluid (Lehtinen and Walsh 2011; Willaredt et al. 2013). It has been shown that during cell division, the ciliary membrane containing signal transducer proteins is endocytosed and associated with the older mother centriole. As a result, the daughter cell that inherits the older mother centriole (the future stem cell) is able to form the primary cilium earlier than the other daughter cell (Paridaen et al. 2013). It has been hypothesized that this process equips one of the daughter cells with signals that maintain its stemness. This notion is supported by the finding that depletion of the ciliary GTPase ADP-ribosylation factor-like protein 13b (Arl13b) reverses the apical–basal polarity of the developing cortex, resulting in neuronal migration toward the ventricles and mitosis at the pial surface (Higginbotham et al. 2013). Thus, several proteins localized to the centrosome in a cell type-specific manner exhibit potent effects on neural stem cell fate, influencing whether they differentiate or remain as stem cells in their niche.

### Microtubules

The microtubule (MT) network is a highly dynamic structure crucial for many cell functions. They are not classically considered to be organelles, but given that MTs represent a significant fraction of the cell mass, one may consider MTs to be nonmembranous organelles. Depending on their composition (i.e., tubulin isoforms), interaction partners, and cell type, MTs assemble into different complexes (Conde and Cáceres 2009; Gudimchuk and Mcintosh 2021). They also associate with distinct RBPs and transcripts encoding proteins involved in MT function, such as spindle formation (Sharp et al. 2008). For example, the MT plus-end-interacting protein adenomatous polyposis coli (APC), known for its role in WNT signaling (Fang and Svitkina 2022), binds mRNAs encoding subunits of MTs (Preitner et al. 2014). In addition to these transcripts, APC binds mRNAs for important developmental factors such as *Tbr1*, *Rbfox2*, and *Pumilio1* (Preitner et al. 2014). In this context, the RBPs Rbfox2 and Pumilio1 are of particular interest, as they regulate the expression of several messenger RNAs (mRNAs) (Jangi et al. 2014; Zhang et al. 2017). The transcripts’ binding to APC suggests that they are transported and potentially locally translated, leading to a local enrichment of these RBPs to regulate specific mRNAs. Future studies are necessary to investigate this aspect of cytoskeleton-mediated RBP regulation. However, recent studies have shown that cytoskeletal elements have the ability to distribute mRNAs and ribosomes within cells, such as cardiomyocytes, due to their transcript binding capacity. Disruption of this transport process leads to mislocalization of nascent proteins around the myonuclei. Therefore, the MT network is necessary for proper RNA and translation hotspot localization (Denes et al. 2021; Scarborough et al. 2021).

The role of MTs in transcript distribution raises the question of how cells select mRNAs for this process. One possibility is the use of RBPs, such as FMRP, discussed above (Ryder et al. 2020). However, RBPs have a broad RNA binding capacity (Schieweck et al. 2021a), making it difficult to select transcripts for specific subcomplexes. This suggests additional selection mechanisms. Interestingly, the RNA and protein exporter Crm1 interacts with the nuclear pore complex (NPC) and the noncentrosomal microtubule-organizing center in yeast (Bao et al. 2018). This finding raises the possibility that the proximity of microtubule-organizing centers to specific NPCs selects transcripts for MT binding and cellular distribution (see below). Notably, the centrosome also is typically close to the nucleus, and several NPC proteins localize to the centrosome during M phase, when the nuclear membrane is dissolved (Chatel and Fahrenkrog 2011). Conversely, some centrosomal proteins, such as Centrin 2, can be part of the nuclear pore complex and be involved in RNA transport (Resendes et al. 2008). These reciprocal moonlighting functions fit the concept of coevolution of centrosomes and nuclear structures and further support the concept of multiplying protein functions by their distinct localization.

Although MTs and their binding proteins, such as APC, are expressed ubiquitously, the levels of their RNA interactors may vary in different subcompartments of a cell. For example, APC binds and localizes β2B-tubulin mRNA to axons (Shigeoka et al. 2016). It is plausible that MT subcomplexes exist that are specialized for axonal transport, while MT complexes residing in the soma exert other functions. Furthermore, the heterogeneity of mRNAs at MTs is supported by transcripts detected at MTs that are expressed in a highly cell type-specific manner, such as *Tbr1*.

### Nuclear pore complexes

The nucleus is the starting point for organelle-mediated RNA inheritance. As the birthplace of RNAs, the nucleus shapes the transcriptome of cells by modulating transcription (Debès et al. 2023), splicing (Furlanis et al. 2019), and 3′-UTR processing (Tushev et al. 2018). Transcripts are exported through NPCs to provide mRNAs to the translation machinery in the cytoplasm. It has been hypothesized that NPCs act as a filter to select certain mRNAs over others for export (Blobel 1985; Fazal et al. 2019). This selection process appears to be driven by specialized structures on NPCs, known as baskets, that assemble with specific mRNAs to mark them for export (Bensidoun et al. 2022). Although NPCs are conserved and ubiquitous organelles in cells, they must adapt to differences in the cellular transcriptome between cell types and differentiation stages to contribute to gene regulation. This notion is supported by pioneering studies that show that the expression levels of NPC components differ between cell types and tissues (Raices and D'Angelo 2012; Kane et al. 2018). Indeed, the differential expression of NPC components is functionally relevant in cancer cells, where their level of expression correlates with tissue-specific malignancies (Borden 2021). In addition, other, noncancerous tissues show that the cell type-specific composition of NPCs matters, as the lack of specific NPC components affects the RNA transport only in specific tissues (Bensidoun et al. 2021). These data demonstrate that NPC heterogeneity affects cell function in a cell type- and tissue type-specific manner. In addition, the interaction of NPCs with the MT network and the centrosome may also enable these organelles to regulate the distribution of transcripts within cells, as discussed above.

### Mitochondria

Mitochondria are the other example of an organelle for which the protein composition has been examined in different cell types, and indeed, profound cell type-specific differences have been observed (Fecher et al. 2019; Russo et al. 2021). Mitochondria are the metabolic hubs of cells (Shen et al. 2022) and hence exhibit a high degree of heterogeneity in function and shape in different cell types (Fig. 1B; Collins et al. 2002). However, their cell type-specific protein composition was not known until recently, due to difficulties in isolating mitochondria in sufficient amounts for comprehensive proteome analysis from specific primary cell types. This can be overcome by growing only one cell type in vitro (Russo et al. 2021) or by the newly developed mitotag approach, expressing an outer membrane protein with a GFP tag in a cell type-specific manner (Fecher et al. 2019). For example, this work showed that astrocytes and neurons differ by ∼20% of their proteome in vitro and in vivo (Fig. 1B; Fecher et al. 2019; Russo et al. 2021). The cell type-specific differences comprise mitochondrial proteins linked to the cell type-specific metabolism, such as fatty acid oxidation specifically in astrocytes, but also an unexpected specificity of antioxidant proteins that are often members of the same family (e.g., MGST); however, different members are expressed in distinct cell types, such as neurons that have higher mitochondrial levels of Mgst3, while mitochondria of astrocytes contain more Mgst1 (Russo et al. 2021). Importantly, this matters at the functional level, as elevating the expression of Mgst3, but not Mgst1, promotes the generation of neurons from astrocytes (Russo et al. 2021). As the same has been observed for several other neuron-enriched mitochondrial proteins (Russo et al. 2021), these data demonstrate that proteome heterogeneity of mitochondria matters for the function and generation of specific cell types.

In addition to their distinct protein composition, mitochondria also interact with specific RBPs such as PUF3 and, ultimately, mRNAs (García-Rodríguez et al. 2007; Fazal et al. 2019; Qin et al. 2021). As a result, translationally active ribosomes have been identified on the outer mitochondrial membrane (OMM) (Lesnik et al. 2014; Gold et al. 2017). An essential function of mitochondrially localized translation is to maintain mitochondrial homeostasis (Zabezhinsky et al. 2016). For instance, the transcript that encodes mitochondrial aminoacyl-tRNA synthetases, IARS2, interacts or closely associates with mitochondria (Fig. 1B; Fazal et al. 2019). Furthermore, translation on mitochondria is needed for controlling axonal mitophagy of damaged mitochondria in neurons, a crucial process in neurodegenerative diseases (Harbauer et al. 2022). The identified transcripts bound by mitochondria encode proteins necessary for maintaining mitochondrial homeostasis. Therefore, these interactions likely occur in almost all cell types. However, RNA localization to mitochondria has a highly specific role in the formation of mitochondrial clusters in oocytes (Cheng et al. 2022). A pioneering study identified the RBP ZAR1 as a promoter of the assembly of the mitochondria-associated ribonucleoprotein domain (MARDO), a prerequisite for mitochondrial clustering in oocytes (Cheng et al. 2022). Although the function of mitochondrial clustering in oocyte maturation remains unknown, it has been suggested that mitochondria cluster near sites that require a higher energy supply (Cheng et al. 2022). Since RNA–mitochondria interaction appears to be required for mitochondrial clustering, it is tempting to speculate that RNA acts as a scaffolding molecule to promote assembly. RNA has been shown to promote biomolecular condensation (Langdon et al. 2018; Roden and Gladfelter 2021), and RNAs can self-assemble into higher-ordered complexes such as stress granules in vitro (Van Treeck et al. 2018). This process is inherently associated with phase separation (Langdon et al. 2018). It has been demonstrated that RNA's secondary structure (Langdon et al. 2018; Bevilacqua et al. 2022) and methylation (Ries et al. 2019) facilitate its phase separation. Although this concept has been applied only to RNA granules such as stress granules or transport granules (Roden and Gladfelter 2021), it is tempting to speculate that RNA interaction with specialized organelles might also provide a scaffold for their assembly. Therefore, organelles might select RNAs not only to modify their own proteome or increase local RNA levels but also to enhance complex formation (Van Treeck et al. 2018). Thus, mRNAs localized and translated at the OMM may also allow efficient assembly of factors required for mitochondrial clustering. This assembly process could be driven by the optimal stoichiometry of interaction partners synthesized locally at the OMM. Such a process has been shown to exist for the assembly of Septin complexes formed on moving endosomes in *Ustilago maydis* (Baumann et al. 2014). Collectively, these studies demonstrate that the crucial cell type-specific functions of mitochondria are mediated by their proteome, RBP, and RNA diversity in different cell types.

While both proteome-wide heterogeneity and its functional relevance have been determined for the above organelles, we now further focus on discussing organelles where this evidence is more patchy and restricted to single proteins or RNA association and their diversity with specific organelles or suborganelles.

### Vesicular sorting organelles

RNA association is not unique to the organelles discussed above but is also found at other organelles (Fig. 1C). In the cytoplasm, transcripts are sorted by different localization signals, most likely embedded in the 3′ UTR (Andreassi and Riccio 2009). This leads to their assembly into free or organelle-associated ribonucleoprotein (RNP) particles, a process that is critically driven by phase separation (Alberti and Dormann 2019). Depending on the function of the encoded proteins, mRNAs are targeted to different organelles. One of the best-characterized examples of RNA–organelle interactions are vesicular sorting organelles such as lysosomes. Lysosomes are critical hotspots for the degradation of extracellular and intracellular proteins in an acidic lumen. The position of lysosomes in cells, such as HeLa cells, and the luminal pH are determined by the ratio of the protein interactor Rab7 and Arl8b (Johnson et al. 2016). In addition, these organelles interact with a variety of proteins such as the mechanistic target of rapamycin (mTOR) (Ratto et al. 2022) to sense amino acid levels or with lysosomal and mitochondrial biogenesis factors (Malik et al. 2023). A seminal study showed that annexin A11 associates with the lysosomal surface and interacts with RNA granules to enable long-distance RNA transport (Liao et al. 2019). Although these lysosome interaction partners are ubiquitously expressed in almost all cells, these findings highlight the functional heterogeneity of lysosomes. It is plausible that RNA and protein interactors vary between compartments, given the differences in soma and axonal as well as dendritic transcriptomes and proteomes (Cajigas et al. 2012; Shigeoka et al. 2016; Biever et al. 2020), suggesting distinct lysosomal interactomes. However, direct evidence for lysosomal heterogeneity is still lacking.

Endosomes are another example of this class of organelles. Endosomes serve as landing platforms for many protein and RNA interactors. One example is the five-subunit endosomal Rab5 and RNA/ribosome intermediary (FERRY) complex that binds to mRNAs and the endosome via Rab5 (Schuhmacher et al. 2023). These endosome-bound mRNAs are translated on endosomes, as these organelles also bind to ribosomes (Fig. 1C; Cioni et al. 2019; Schuhmacher et al. 2023). Interestingly, transcripts found at endosomes encode mitochondrial proteins (Schuhmacher et al. 2023). As a result, the interaction between endosomes loaded with these mRNAs and mitochondria is important for the function of mitochondria in the axon (Cioni et al. 2019). The role of endosomes in the distribution of mRNAs is conserved from rodents to *Xenopus* to fungi such as *U. maydis* (Higuchi et al. 2014; Cioni et al. 2019; Schuhmacher et al. 2023). This underscores their importance in maintaining cellular homeostasis and growth. Coupling mRNA transport to endosomes is an elegant way to synchronize cellular resources required for growth. Whether it is the growing tip of a fungus or the branching point of axons in neurons, all these processes require lipids, energy, and the synthesis of new proteins. Endosomes are known to provide lipids as well as mRNAs and the translation machinery. Hence, it is plausible that specialized endosomes serve as “construction platforms” to remodel cellular environments locally.

Although these findings imply heterogeneity in endosome function within cells, it is now important to perform unbiased proteome analysis of endosomes in different cell types to examine to what extent endosomes differ in function and/or composition between cell types and stages.

### Nucleoli

Nucleoli are membrane-less organelles in the nucleus, important for translation (Lafontaine et al. 2021). Given this pan-cellular function, so far no efforts have been made to examine their composition in a cell type-specific context, even though dynamic shuttling between nucleolar proteins and stress granules has been described (Qin et al. 2023). However, one protein associated with nucleoli in a highly cell type-specific manner has been shown to have profound functional effects, highlighting the need to profile this compartment as well. Tmf-regulated protein 1 (Trnp1) is enriched at the outer surface of the nucleolus in self-renewing neural stem cells but not differentiating neural stem cells (Esgleas et al. 2020). It regulates phase transition and nucleolar size and function (Esgleas et al. 2020). This has profound effects on neural stem cell self-renewal, the size of a brain region, and folding in ontogeny and phylogeny (Stahl et al. 2013; Martínez-Martínez et al. 2016; Kliesmete et al. 2023). These observations call for unbiased analysis of nucleolus composition, given its profound role in regulating translation and cell cycle length, key processes regulating stem cell self-renewal, differentiation, and *trans*-differentiation (Camargo Ortega and Götz 2022; Sonsalla et al. 2024).

### Ribosomes

The examples discussed thus far impressively demonstrate the enormous heterogeneity that exists in the protein composition, interaction partners, RNA association, and cellular localization of pan-cellular organelles (Fig. 1D). It is interesting to note that this concept can be applied even to suborganelles, such as ribosomes. Historically, ribosomes have been regarded as molecular machines that produce proteins upon activation. However, pioneering studies have shown that ribosomes exhibit heterogeneity, enabling them to selectively translate mRNAs (Xue and Barna 2012). Ribosomal proteins play a central role in the regulation of translation in this context. For instance, Rpl13a, which is released from ribosomes and binds with particular transcripts to prevent their translation, represents one such example (Mazumder et al. 2003). Although the extraribosomal function of Rpl13a may be independent of ribosome heterogeneity, it is possible that the ribosome serves as a storage site for Rpl13a to control translation inhibition. In light of the clear evidence for local translation at the centrosome (for review, see Zein-Sabatto and Lerit 2021), it is of interest that Rpl13a localizes specifically to the centrosomes of neurons but not neural stem cells (O'Neill et al. 2022). Notably, many more ribosomal proteins associate with the neuronal centrosomes than with the neural stem cell centrosomes, comprising those that inhibit or promote translation. One example is Rps25, which is essential for noncanonical, repeat-associated non-AUG (RAN) translation of *C9orf72*, which results in the generation of dipeptide repeat proteins (Fig. 1D; Yamada et al. 2019). The association of these ribosomal proteins with neuronal centrosomes implies a regulatory hub. Because ribosomal proteins have an impact on the translation of selective transcripts, it is conceivable that their binding to neuronal centrosomes directs the local translation of specific transcripts. During development, Rpl38 has been proposed to regulate the translation of specific Hox mRNAs by facilitating the formation of active ribosomes on these transcripts (Fig. 1D; Kondrashov et al. 2011). Importantly, this finding has been challenged by recent studies (Akirtava et al. 2022; Ivanov et al. 2022) that question a direct regulatory role of Rpl38 in selective Hox gene expression. Notably, Rpl38 is selectively bound to NSCs but not to neuronal centrosomes (O'Neill et al. 2022), suggesting a possible role in regulating gene expression in the daughter cells and eventually an effect on cell fate commitment. These examples demonstrate how ribosomal proteins may actively regulate the translation of specific mRNAs. However, future experiments are clearly needed to unravel their role in active translation control.

Ribosomal proteins can be produced and exchanged locally (Shigeoka et al. 2019; Fusco et al. 2021). Among the ∼79 ribosomal proteins, those that reside on the ribosomal surface are preferentially exchanged (Shigeoka et al. 2019; Fusco et al. 2021). It is thus conceivable that there are specialized ribosomes guided by different ribosomal protein compositions that select some mRNAs for translation. This idea is supported by the discovery that not all ribosomal proteins are found on ribosomes. Rpl10a, Rpl38, Rps7, and Rps25 have been identified as variable ribosomal proteins, which bind to some but not all ribosomes (Shi et al. 2017). Furthermore, ribosomes containing Rps25 or Rpl10a regulate the translation of different transcripts (Fig. 1D; Shi et al. 2017). Also, ribosome-associated proteins (RAPs) define specialized ribosomes. One of these RAPs is the metabolic enzyme pyruvate kinase muscle (PKM). PKM is a noncanonical RNA-binding protein that selects mRNAs for ER-localized translation (Simsek et al. 2017). These examples illustrate the heterogeneous nature of ribosomes regarding their interaction partners.

In addition to the protein composition, the rRNA component of ribosomes also exhibits some degree of specificity. Although the general building blocks of ribosomes are relatively conserved during evolution, they have undergone significant changes (Xue and Barna 2012). The majority of variation is found in the expansion segments, which are variable regions that have tentacle-like rRNA structures. The expansion segments are ribosome adaptations that enable efficient translation of specific mRNAs, including Hox mRNAs (Leppek et al. 2020), through internal ribosome entry sites (IRESs). While the relevance of these IRESs in regulating mRNA translation is still under debate (Akirtava et al. 2022), these findings suggest that ribosomes have been adapted during evolution to efficiently translate specific transcripts. This implies another layer of ribosomal heterogeneity between species. Besides expansion segments, ribosomes can enhance their diversity through modifications. Ribosomes’ 2′-O position of the ribose moiety is subjected to heavy methylation (Gay et al. 2022). These modifications are crucial for translational fidelity and activity (Jansson et al. 2021; Khoshnevis et al. 2022). The dynamic regulation of such rRNA methylation occurs in a region-specific manner in brain development and affects neuronal differentiation by modulating ribosome association with FMRP (Häfner et al. 2023). Overall, these findings reveal the direct function of ribosomes in controlling the translation of a specific subset of transcripts, emphasizing the crucial impact of ribosome heterogeneity on cellular homeostasis and plasticity, including in development.

## Regulation of organellar heterogeneity

**Figure 2.**
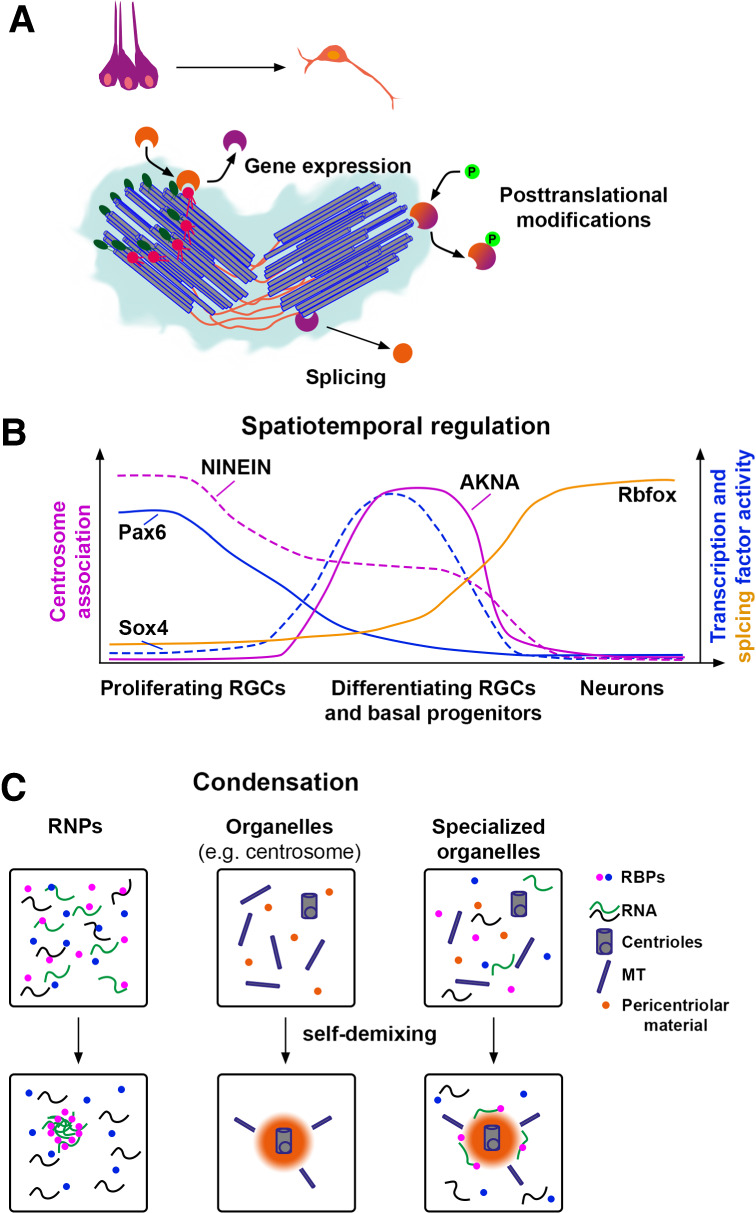
Regulation of organellar heterogeneity. (*A*) Overview of possible regulatory pathways affecting centrosome binding. (*B*) Organellar heterogeneity can be regulated by changes in expression and splicing during differentiation. (*C*) RBPs and RNA have an inherent tendency to self-demix and to condense in the cytosol. Some organelles, including centrosomes, undergo a similar assembly process. Based on these findings, it is plausible that the cocondensation of organelles, proteins, and RNA interactors is responsible for the formation of specialized organelles.

The results described above clearly show that pan-cellular organelles display an enormous degree of heterogeneity that regulates different aspects of cellular homeostasis and plasticity. This prompts the important question of how this heterogeneity is established and controlled. Pioneering studies have unraveled that gene expression, post-translational modifications, and alternative splicing can be major regulators of organellar interactions (Fig. 2A).

Localization of a protein to a certain organelle in a cell type-specific manner might be a result of differential gene expression. For example, this is the case for Akna, which is transcriptionally up-regulated by the transcription factor Sox4 in differentiating neural stem cells and during epithelial–mesenchymal transition in other epithelial cells and hence is found only in these at the centrosome (Fig. 2A,B; Camargo Ortega et al. 2019). In addition, Akna localization at the centrosome is regulated by post-translational mechanisms such as phosphorylation changes during mitosis, when it dissipates from the centrosome like many other centrosome proteins (Fig. 2A; Camargo Ortega and Götz 2022). This is also the likely mechanism of localizing ubiquitous proteins present in a single isoform, such as PRPF6, to the centrosome in some cell types but not in others. This brings us to differential splicing as another possibility to localize a protein to a specific site; namely, to include specific protein domains for interaction by differential splicing. This is the case for Ninein, which associates with the centrosome in self-renewing neural stem cells but not in neurons due to differential splicing (Fig. 2A,B; Zhang et al. 2016). Indeed, the expression of some splicing factors is restricted to specific developmental stages (Raj and Blencowe 2015; Zhang et al. 2016).

Another phenomenon to consider in this context is self-demixing of protein and RNA complexes. It has been shown that some proteins and RNAs have the ability to phase-separate from their liquid environment and form distinct complexes. Originally described for RNP particles, the purpose of self-demixing is to buffer transcriptional and translational fluctuations in cells (Bauer et al. 2022; Cardona et al. 2023). Importantly, some organelles also undergo liquid–liquid phase separation, such as the nuclear pore complex (Celetti et al. 2019), nucleolus (Lafontaine et al. 2021), or centrosome (Woodruff et al. 2017), although phase separation of the latter is still under debate (Raff 2019). Even membranous organelles such as mitochondria rely to some degree on phase separation (Long et al. 2021). Based on these results, it is conceivable that phase separation and self-demixing may contribute to the establishment of organellar heterogeneity. In this scenario, certain proteins and RNA have the intrinsic ability to self-demix from the cellular cytosolic environment along with the organelles with which they interact (Fig. 2C). This allows organelles to diversify. The degree of association may be influenced by the expression levels of these proteins and RNAs, as has been shown for processing bodies (Cardona et al. 2023). This model could potentially explain the differences in organelle composition between various cell types and developmental stages. Each cell type has a unique transcriptome and proteome, which may result in different RNA and protein interactors of organelles demixing and condensing by phase transition, which could contribute to the distinct functions of organelles in different cell types.

An important task for future studies is to elucidate the mechanisms regulating the formation of organellar heterogeneity and determine which of the possibilities discussed above may be prevalent for specific organelles or whether there is a predominant upstream regulatory process of organellar heterogeneity. Importantly, organellar heterogeneity may also be subject to profound remodeling; for example, by the highly dynamic trafficking of proteins between organelles (Qin et al. 2023). This is of crucial relevance given the functional importance of organellar heterogeneity.

## Functions of organellar heterogeneity

### Multiplying functional diversity of proteins

Beyond the specific function of an organelle in its respective cell type-specific context, organellar heterogeneity may contribute to multiplying protein functions by recruitment to different organelles. Mammals generate a disproportionate cell diversity relative to their genome size, which is not significantly different from that of, for example, *Caenorhabditis elegans*. One way to achieve such diversity with the same number of genes is to multiply protein function by using the same proteins in different contexts. This is nicely illustrated by moonlighting functions of proteins that play different roles at distinct positions in a cell (Singh and Bhalla 2020; Somma et al. 2020). For example, NPC proteins regulate import/export to the nucleus in G1 phase and spindle assembly during M phase, when the nuclear membrane is dissolved (Guglielmi et al. 2020). Likewise, many transcription factors and chromatin remodelers localize to the midbody and play a role in adequately separating the daughter cells after mitosis. Kinetochore proteins play a role in insulin signaling (Singh and Bhalla 2020). Proteins with moonlighting functions are listed in databases such as MoonProt or multitaskProtDB, which comprise close to 1000 proteins, even though many of the newly discovered proteins with multiple functions, such as PRPF6, are not yet listed there. Particularly relevant to our discussion is that the alteration of protein localization is one of the mechanisms contributing to evolution of multifunctional proteins (Singh and Bhalla 2020). Thus, distinct localization of a protein in a cell may diversify its function and specify the function of the respective organelle in a cell type- or context-specific manner. Consequently, the moonlighting functions of specific proteins may contribute to organellar heterogeneity.

### Inheritance of heterogeneous organelles

Another function of heterogeneous organelles is their asymmetric distribution. Cell division requires the equal inheritance of organelles to both daughter cells. This process is conserved across all life forms (Camus et al. 2022). Nevertheless, some organelles show asymmetric inheritance to the next generation of cells. A classic example is oocyte mitochondrial inheritance. These organelles are inherited through the maternal lineage (Wallace 2018). Although the contribution of paternal inheritance has been proposed (Gyllensten et al. 1991; Luo et al. 2018), these findings have been contested by others (Pagnamenta et al. 2021). In other cells, mitochondria are inherited by both daughter cells, but different mitochondria can be asymmetrically inherited. For example, a stem cell often divides asymmetrically, with one daughter cell self-renewing (i.e., remaining a stem cell) while the other daughter cell differentiates. Intriguingly, newly produced mitochondria are inherited by the daughter cell that remains a stem cell, and this is indeed important to maintaining the stemness of this daughter cell (Katajisto et al. 2015). Notably, different age groups of mitochondria transfer unique metabolic profiles to their offspring cells and are therefore significant determinants of cell fate (Döhla et al. 2022). Moreover, older mitochondria may be damaged to some extent, which matters less in the differentiating daughters, as they can be replaced and are often short-lived, while adult stem cells often self-renew and survive for the entire life of the organism.

Apart from mitochondria, other organelles like centrosomes can be inherited asymmetrically, thus defining the cellular status of the descendants (Kahneman et al. 2007). In the developing vertebrate brain, most cells that inherit new mother centrioles leave the ventricular stem cell niche to become differentiating progenies (Wang et al. 2009). In contrast, cells with old mother centrioles remain in the ventricular zone and maintain their original stemness. The variability of centrosomes in neural stem cells, especially the association with RBPs (O'Neill et al. 2022), suggests that these RBPs may also be asymmetrically inherited. This process could potentially prepare cells for differentiation or the maintenance of stemness, as has been shown for the RBP Staufen2 (Stau2). In this case, Stau2's uneven segregation is crucial for balancing the maintenance of neural stem cells and differentiation (Kusek et al. 2012). Importantly, spindles contain different RNAs in the presence or absence of Staufen (Hassine et al. 2020), supporting the concept that RBPs that are asymmetrically inherited bring along their specific RNAs that can be translated quickly and influence the fate of the daughter cell (Li et al. 1997). In this regard, it is also relevant to mention the asymmetric inheritance of the basal process of neural stem cells in the murine cortex (Shitamukai et al. 2011) and the specific localization of RNAs in the basal endfeet of these processes (Pilaz et al. 2016). As organelles are also specifically distributed in these processes and contain different RBPs, organellar heterogeneity forms a platform for the distribution of cell fate determinant proteins, RBPs, and RNAs to specify the progeny cells. Indeed, the uneven inheritance of organelles by progeny appears to be a widespread concept applicable to further organelles. Seminal studies have revealed that lysosomes are also asymmetrically distributed to neural and hematopoietic progenies, thereby contributing to diverse signaling pathways in daughter cells (Bohl et al. 2022; Loeffler et al. 2022). As lysosomes are ubiquitous organelles, it is tempting to speculate that these organelles are specialized to function as signaling transducers. Their interaction with RNA granules supports this notion (Liao et al. 2019). Overall, these findings strongly suggest that the asymmetric inheritance of organelles is a critical determinant of cell fate. However, as evidence exists so far only for some organelles in few cell types, these exciting data call for much more analysis of the role of organelle heterogeneity in asymmetric inheritance.

### Hypothesis: organelle-specific protein translation and folding

We suggest addressing organelle-specific protein translation and folding in the future. As mentioned above, organelles bind and transport a significant portion of the transcriptome. At some organelles, such as mitochondria and centrosomes, these transcripts are translated locally. Recent data have shown that the local transcriptome and, ultimately, the translatome differ between organelles and the cytosol (Fazal et al. 2019). It is important to note that transcripts influence protein synthesis through different mechanisms (Schieweck et al. 2016). For example, the codon usage of transcripts can influence ribosome speed—and thus the translational output—through the corresponding tRNA level (Schieweck et al. 2016; Kirchner et al. 2017). In the case of specialized organelles, the codon usage of the bound mRNAs might be different compared with the cytosol. A recent study supports this idea by demonstrating that transcripts encoding for membrane proteins contain nonoptimal codons to regulate ribosome speed in the process of targeting these proteins to membranes (Pechmann et al. 2014). Adaptations in translation speed may occur preferentially at specialized organelles that bind mRNAs encoding membrane proteins, such as mitochondria (Fazal et al. 2019) or the endoplasmic reticulum (Jan et al. 2014). In addition, ribosome speed depends on the availability of translation factors (Schieweck et al. 2023; Popper et al. 2024). A recent study showed that the translation factor eEF1A2, which affects ribosomal speed, binds to the actin cytoskeleton and regulates its dynamics (Mendoza et al. 2021). In addition, specific translation initiation factors are enriched at centrosomes of neural stem cells and others at the centrosomes of neurons (O'Neill et al. 2022). This suggests that essential translation factors are concentrated at specific organelles, thereby influencing ribosome speed. This may be particularly important during differentiation, when cells alter ribosome speed (Popper et al. 2024). Although direct evidence is largely lacking, it is conceivable that the ribosome speed and translational output differ at organelles. Importantly, ribosome speed affects cotranslational folding trajectories, which can alter the structure and specificity of proteins, as has been shown for the multidrug resistance 1 (MDR1) gene (Kimchi-Sarfaty et al. 2007) or the cystic fibrosis transmembrane conductance regulator (CFTR) (Kirchner et al. 2017; Rauscher et al. 2021). Given the differences in organellar transcriptomes and translatomes, it is conceivable that cotranslational folding is also regulated in an organelle-specific manner. It is possible that this represents another level of organellar heterogeneity, where local synthesis of proteins could influence their structure and thus their specificity.

## The role of organellar heterogeneity in disease

Given the important functions of organellar heterogeneity, its implication in disease may not be surprising, but the scope of its importance in this regard may well be. One of the best-characterized organelles with a causative link to clinically relevant diseases is the centrosome. Many centrosome proteins have been implicated in neurodevelopmental disorders (Remo et al. 2020; Farcy et al. 2023), but the organ and cell type specificity is mostly overlooked. For example, Aspm is a ubiquitous centrosome and spindle protein important in mitosis, and hence it may not be surprising that its mutation has been implicated in microcephaly (Passemard et al. 2016; Garrett et al. 2020). However, it is not clear why loss of Aspm causes no phenotype in most other organs that are likewise generated by dividing cells, except the germ cells and the brain (Pulvers et al. 2010). Clearly, centrosome heterogeneity (namely, its specific composition in cells of different organs, tissues, and species) may well be at the bottom of the organ specificity of this (and many other) mutations. This concept has been recently highlighted by the mutation of the ubiquitous splicing protein PRPF6 that leads only to neurodevelopmental defects despite its presence in all cell types. The organ-specific function of PRPF6 at the centrosome discussed above prompts the suggestion that organ-specific localizations of ubiquitous proteins lead to organ-specific functions that are disturbed only in these organs upon mutation or dysfunction of the respective proteins (O'Neill et al. 2022). Importantly, the centrosome proteomes of different cell types show significant overlap with gene variants of only specific neurodevelopmental and neuropsychiatric disorders, further supporting the concept that the specific composition of the centrosome affects distinct processes in a cell type-specific manner (O'Neill et al. 2022). This concept is also evident in ciliopathies, diseases caused by dysfunction or absence of cilia (Mill et al. 2023). While there are common phenotypes observed in ciliopathies, there are also mutations that result in organ- and brain-specific deficits (Lovera and Lüders 2021; Mill et al. 2023). For example, deletion of the ciliary protein Arl13b reverses the polarity of the cortical wall (Higginbotham et al. 2013). Notably, Arl13b mutations have been found in patients with Joubert syndrome (Caspary et al. 2007; Cantagrel et al. 2008), a neurodevelopmental disorder characterized by malformation of the brainstem and the absence or underdevelopment of the cerebellar vermis (Brancati et al. 2010). However, it is not known why specifically these brain regions are affected even though all cells have cilia and all stem and progenitor cells in the brain require cilia to perceive certain signaling pathways.

In addition to the centrosome, mutations of proteins located at other organelles have also been implicated in organ- and brain-specific disorders. For example, mutations in Rab7a, an endosome interactor, have been found in patients with Charcot–Marie–Tooth type 2B disease (Cherry et al. 2013), a neuropathy characterized by axonal dysfunction and degeneration. Interestingly, as endosomes serve as hotspots for mitochondrial protein synthesis, Rab7a mutation impairs axonal protein synthesis and mitochondrial function as well as axonal viability (Cioni et al. 2019), suggesting a direct link between specialized transport endosomes and the disease. The importance of vesicles as transport vehicles for mRNAs is highlighted by a recent study describing mutations found in amyloid lateral sclerosis patients that impair the ability of annexin A11 to link RNA granules to moving lysosomes (Liao et al. 2019).

Another impressive example for cell type specificity of mutations that impair ubiquitously expressed proteins is the blood disorder Diamond–Blackfan anemia (DBA). Patients with DBA show a selective reduction of erythroid precursors and progenitors, while all other lineages are normally produced (Nathan et al. 1978; Ohene-abuakwa et al. 2005). At the genetic level, DBA patients preferentially have loss-of-function mutations in ribosomal protein genes (Mirabello et al. 2017). Ribosomal proteins are expressed throughout the body, but in DBA patients their loss of function affects only erythroid but not other hematopoietic lineages due to impaired translational control (Khajuria et al. 2018). A similar pathomechanism has been proposed for the 5q syndrome, a myelodysplastic syndrome caused by haploinsufficiency of RPS14 (Narla and Ebert 2010). Mutations in ribosome biogenesis factors can cause various syndromes, including Shwachman–Diamond syndrome, X-linked dyskeratosis congenita, cartilage hair hypoplasia–anauxetic dysplasia (CHH-AD), and Treacher Collins syndrome (TCS) (Narla and Ebert 2010; Kang et al. 2021). These syndromes are characterized by bone marrow failures and exhibit a surprising tissue specificity. These findings indicate the presence of specialized ribosomes that regulate translation in specific cells or tissues and are preferentially impacted by the mutations discovered in these patients. Another, not mutually exclusive, explanation is that the quantity of ribosomes affects certain transcripts more than others due to differences in initiation, recycling, and rescuing rates. In this scenario, specific mRNAs, particularly poorly initiated mRNAs, are more vulnerable to a decrease in ribosome levels (Mills and Green 2017). This may explain the cell and tissue selectivity of the aforementioned diseases.

Given the above, much more research on organellar heterogeneity is needed to tackle some of the biggest questions in pathology: Why are specific cell types particularly vulnerable upon dysfunction of pan-cellular organelles? This is also the case in neurodegenerative diseases for which mitochondrial dysfunction is a common denominator, yet only specific neuronal subtypes are affected even differentially for different mitochondrial protein mutations. Thus, organellar heterogeneity may hold the key to answering some of the most pressing and general questions in human health and disease.

## Outlook—how to study and change organellar heterogeneity

Given the functional and disease relevance of organellar heterogeneity discussed above, it is of crucial importance to characterize organellar composition of proteins and RNAs more comprehensively, as in many cases we know this only for one or a few cell types. The purpose of this review is to overcome one of the biggest hurdles; namely, the prevailing concept of the similarity of pan-cellular organelles in different cell types. For example, this has led to the absence of any centrosome proteomes from neural cell types until recently (O'Neill et al. 2022), even though centrosome proteomes have been generated long ago. Excitingly, the development of human iPSCs and robust protocols for differentiation into many human cell types now allow the generation of sufficient material of almost all human cell types, even for biochemical approaches that require a lot of material. In addition, organellar proteomics allow the enrichment of specific organelles in distinct fractions (Itzhak et al. 2018; Schessner et al. 2023), theoretically enabling the observation of shifts of proteins between organelles. However, this approaches its limits when cell types are very different. A beautiful approach to monitor the shift of proteins between organelles even at single-cell resolution is the tagging by a fluorescent protein followed by multicontent imaging (Qin et al. 2023). Such an approach in human iPSCs would be a fantastic resource to examine not only pure populations of specific human cell types but also organoids or cell mixtures to understand how cell–cell interactions may affect organellar heterogeneity.

Nevertheless in vitro approaches have their limitations, and for analysis in tissue sections spatial transcriptomics and proteomics may be very useful, especially with improved single-cell resolution. Applying these techniques on complex-shaped cells, such as neurons, provides spatial information on mRNA localization (Perez et al. 2021). Similarly, the isolation of endfeet from radial glial cells resident in the developing brain allows the study of the local transcriptome (Pilaz et al. 2016). In combination with single-cell ribosome profiling approaches (Vaninsberghe et al. 2021; Ozadam et al. 2023) and single-cell proteomics (Brunner et al. 2022; Mund et al. 2022), it is possible to identify stoichiometric imbalances of organellar building blocks that might indicate the existence of specialized organelles, as demonstrated for proteasomes (Sun et al. 2023) or ribosomes (Shi et al. 2017). To investigate organellar specialization, other methods are needed that provide higher spatial resolution. One of these methods is the proximity biotinylation assay. In this methodology, established organellar interactor proteins are merged with the peroxidase APEX to attach biotin to protein (Markmiller et al. 2018) and RNA interactors (Fazal et al. 2019). By using split-APEX fusion constructs (Han et al. 2019) alongside these strategies, specific protein and RNA interactors for the organelle can be determined. Furthermore, proximity biotinylation assays exploiting APEX and TurboID can be used to study protein transport between organelles (Qin et al. 2023). Apart from these methods, the separation of fluorescently labeled single particles (Hubstenberger et al. 2017) and, ultimately, organelles is also an effective way to identify interactor partners in an unbiased manner. In addition, advanced microscopy approaches such as DNA-PAINT allow for multiplexed detection of proteins within cells (Jungmann et al. 2014).

Functional assays are necessary to establish the link between the existence of specialized organelles and their specific functions within cells. One such assay involves transplanting organelles into recipient cells. Although mitochondrial (Sercel et al. 2021) and centrosome (Tournier et al. 1989) transplantations have been demonstrated, additional efforts may be needed to ensure the reproducible uptake into various cell types. It is worth considering that other organelles may be amenable to this technique. Given the important functions and disease relevance of organellar heterogeneity identified only from our yet very limited knowledge about this phenomenon, future studies are essential to identify the protein and RNA interactomes of different organelles in different cell types. This will pave the way toward a better understanding of multiplying protein functions in ontogeny and phylogeny, of how organellar functions can be further specified to serve the needs of distinct cell types, and their relevance to cell type- and organ-specific disease etiology.
